# In Vitro Modelling of Obstructive Sleep Apnea by Intermittent Hypoxia of Human Embryonic Stem Cell-Derived Cardiomyocytes: Expression of ERK1/2, ERK5 and Erbin

**DOI:** 10.3390/ijms27156804

**Published:** 2026-07-29

**Authors:** Danielle Regev, Sharon Etzion, Aviv Goldbart, Jacob Gopas

**Affiliations:** 1 Shraga Segal Department of Microbiology, Immunology and Genetics, Faculty of Health Sciences, Ben-Gurion University of the Negev, Beer-Sheva 8410501, Israel; danirege@post.bgu.ac.il; 2 Regenerative Medicine & Stem Cell Research Center, Ben-Gurion University of the Negev, Beer-Sheva 8410501, Israel; sharetz@gmail.com; 3 Soroka University Medical Center, Department of Pediatrics, Faculty of Health Sciences, Ben-Gurion University of the Negev, Beer Sheva 8410501, Israel; avivgold@bgu.ac.il; 4 Pediatric Pulmonary and Sleep Research Laboratory, Faculty of Health Sciences, Ben-Gurion University of the Negev, Beer Sheva 8410501, Israel

**Keywords:** obstructive sleep apnea, intermittent hypoxia, hESC-CM, human embryonic stem cells derived cardiomyocytes, Erbin, ERK1/2, ERK5

## Abstract

Obstructive sleep apnea (OSA) syndrome is characterized by repetitive nocturnal airway obstruction and is associated with intermittent hypoxia (IH). The leading cause of death among OSA patients is cardiovascular morbidity, which is greatly enhanced by IH. Despite the existence of standard treatment, cardiovascular morbidity remains unaddressed. Given the central role of IH in OSA-related cardiac damage, the present study aimed to elucidate the mechanisms underlying IH-induced cardiac injury in order to better understand and potentially improve upon current therapeutic approaches. Using human embryonic stem cell-derived cardiomyocytes (hESC-CMs) as a novel in vitro model, IH was successfully induced, and its effects on key signaling pathways were investigated. Following IH exposure, significant activation of ERK1/2, ERK5, and Erbin was demonstrated. Notably, the concurrent increase in both ERK1/2 activation and Erbin expression following IH suggests a more complex regulatory relationship between these molecules than previously appreciated. Furthermore, pathway-specific inhibition of ERK1/2 and ERK5 attenuated the IH-induced decline in beating rate, with significant restoration, following normoxic recovery. This study provides an innovative approach for in vitro investigation of OSA-associated cardiovascular morbidity and supports the search for novel pharmacological agents and molecular targets to improve the diagnosis and treatment of affected patients.

## 1. Introduction

Obstructive sleep apnea (OSA) syndrome is the most prevalent sleep-related breathing disorder [1]. It is characterized by a collapse of the pharynx during sleep and repetitive nocturnal upper airway obstructive events associated with intermittent hypoxia (IH) [2].

OSA has been estimated to affect 15–24% of adults and 10–20% of pediatrics patients [3], yet it is often left underdiagnosed and untreated [4,5]. The epidemiology of OSA varies greatly, with 5% to 61% in women and 14% to 84% in men [5].

OSA has a wide array of morbidities, such as neurocognitive, pulmonary, and cardiovascular implications. Cardiovascular morbidity is considered to be the leading cause of death from OSA. It encompasses many morbidities such as hypertension, coronary artery disease, heart failure, ventricular hypertrophy, and myocardial ischemia [6,7]. Hypoxia severity and burden have recently been shown to be a strong predictor of cardiovascular and mortality in adult patients [8].

Continuous Positive Airway Pressure (CPAP) is the standard treatment for Obstructive Sleep Apnea (OSA). Although CPAP restores respiration and sleep architecture, several randomized controlled trials (RCTs) and meta-analyses have reported no risk reduction in adverse cardiovascular events from the use of CPAP therapy in OSA [9,10].

Our laboratory has previously shown that there is a local Nuclear-factor kappa-light-chain-enhancer of activated B cells (NF-κB) activation and Interleukin 1-alpha (IL-1α) expression in adenoid and tonsillar tissue of children with OSA [11], as well as activation of NF-κB in cells incubated with sera of these children [12]. We also were able to show for the first time the effect of sera from OSA patients on beating human cardiomyocytes differentiated from human embryonic stem cells (hESC-CMs) [13]. We showed NF-κB activation as well as a decrease in beating rate, contraction amplitude, and a decrease in calcium signaling [13]. In our recent publication, we subjected hESC-CMs to IH conditions, thus mimicking IH repetitive obstructive events in patients. At first, we showed the ability of our in vitro system to induce IH. Next, we showed a decrease in beating rate as well as a decrease in contraction strength. Furthermore, we showed NF-κB activation with an increase in p65 peri-nuclear staining, which points to NF-κB activation as well, following IH. Finally, we measured the expression of various secreted molecules in the conditioned media (CM) of the cells, following IH. Several proteins were detected, all of which exhibited pro-inflammatory properties and have been previously associated with OSA [14].

Various signaling pathways are associated with OSA, hypoxia, or cardiovascular diseases. Among them, Extracellular signal-related kinase 1/2 (ERK1/2) emerged as a key signaling pathway in cardiac damage. ERK1/2 is a dual phosphorylated kinase that belongs to the mitogen-activated protein kinase (MAPK) pathway. Once activated, ERK1/2 can induce several processes in cardiomyocytes, such as apoptosis, cell survival, protein synthesis, and hypertrophy [15,16,17]. ERK1/2 has been linked to pathological hypertrophy, an initial step towards cardiac damage, specifically when it phosphorylates nuclear targets such as E twenty-six (ETS)-like 1 transcription factor (Elk1), mitogen- and stress-activated protein kinase 1 (MSK1), and c-Myc [15]. In pathological hypertrophy, ERK1/2 can lead to the synthesis and activation of pro-hypertrophic proteins such as ANP and transcription factors such as GATA4 [17].

Erbin (Erbb2 interacting protein, ERBB2IP) is a protein capable of inhibiting several signaling pathways, among them ERK1/2. Erbin inhibits ERK1/2 phosphorylation and, therefore, activation by binding to Soc-2 suppressor of clear homolog (Shoc2) and preventing ERK1/2 phosphorylation through MEK1/2, ERK1/2 has been related to hypertrophy, and Erbin might be able to suppress it [18,19]. Extracellular signal-related kinase 5 (ERK5), is another member of the MAPK pathway. ERK5 is activated through dual phosphorylation, which reveals a C-terminal nuclear localization signal. ERK5 has been associated with the proliferation of cancer cells, bone mineralization, inflammation, embryonic cardiac development, and hypertrophic-related processes such as serial sarcomere assembly, impaired contraction, and increased apoptosis [20]. ERK5 activation showed an increase in inflammatory molecules such as interleukin-1β (IL-1β), stromal cell-derived factor 1 (SDF-1), and monocyte chemoattractant protein 1 (MCP-1), triggering hypertrophy [21]. ERK5 may be another key signaling pathway involved in cardiac impairment in OSA.

Considering IH as a key feature of OSA and activation of the NF-κB pro-inflammatory pathway [14] it is of much interest to understand the interplay of these factors and their effect on cardiac damage in OSA. In order to study the detrimental effect of cyclical IH on cardiomyocytes, we established a unique in vitro system not previously utilized in OSA research. Our system mimics the pattern of IH in patients on hESC-CMs. We measured different biochemical and cellular parameters following exposure to hypoxia or normoxia. By understanding the mechanism(s) of cardiovascular morbidity in OSA, we may be able to prevent cardiac damage. We decided to concentrate our efforts on a few promising signaling pathways to better understand their role in cardiac damage in OSA.

## 2. Results

### 2.1. Erbin and ERK1/2 Phosphorylation in hESC CMs Following IH

**Figure 1:**
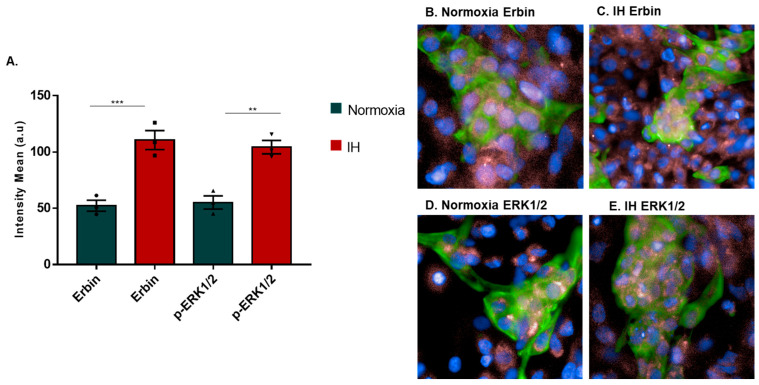
Increase in Erbin expression and ERK1/2 phosphorylation in hESC-CMs following IH: (**A**) quantification of Erbin and p-ERK1/2 following normoxia or IH only on CMs. Normoxia (21% O_2_, 37 °C), intermittent hypoxia (IH) (1% O_2_, 37 °C) for 12 h (60 Cycles). (**B**–**E**) immunostaining of Erbin, p-ERK1/2, and cardiac troponin T (cardiomyocytes’ specific marker) following normoxia or IH. Differentiated CMs were detected with antibodies to cardiac troponin T (green) and Erbin or p-ERK1/2 (pink), followed by the appropriate secondary antibody. Nuclei were stained with DAPI (blue); (**B**) normoxia conditions and Erbin; (**C**) IH conditions and Erbin; (**D**) normoxia conditions and p-ERK1/2; and (**E**) IH conditions and p-ERK1/2. Results are averages of 3 separate experiments performed in 10 replicates. ANOVA analysis ** *p* < 0.01, *** *p* < 0.001; 40× magnification.

Cardiac hypertrophy is a key step towards the development of various cardiovascular diseases, such as heart failure and arrhythmias [7]. Hypertrophy is highly prevalent among OSA patients as well [8]. Although many signaling pathways are involved in cardiac hypertrophy, ERK1/2 signaling is best studied [15]. Another protein that is involved in ERK1/2 signaling is Erbin. Erbin has a dual role regarding ERK1/2 activation. Erbin can modulate ERK1/2 signal and possibly induce hypertrophy [19]; therefore, we determined whether IH affects Erbin expression and ERK1/2 phosphorylation (p-ERK1/2). Our results showed that IH increases both Erbin expression and ERK1/2 phosphorylation (Figure 1A). An increase of both Erbin expression and ERK1/2 phosphorylation is consistent with the possible involvement of IH in sleep apnea-related hypertrophy.

### 2.2. ERK1/2 and ERK5 Phosphorylation in hESC CMs Following IH

**Figure 2:**
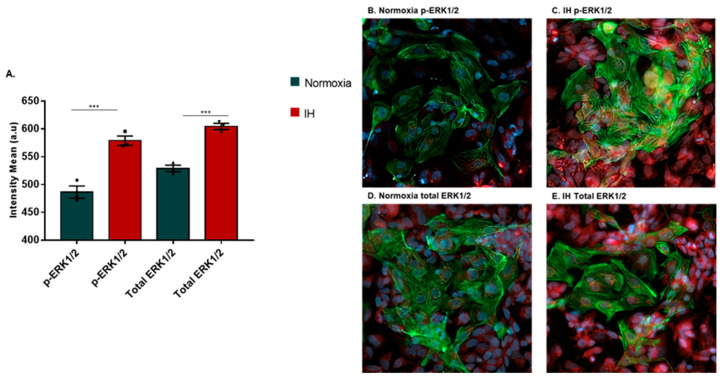
Changes in phospho ERK1/2 and Total ERK1/2 expression in hESC-CMs following IH: (**A**) Quantification of phospho-ERK1/2 (p-ERK1/2) and total ERK1/2 expression following Normoxia or IH. (**B**–**E**) immunostaining of p-ERK1/2, Total ERK1/2, and cardiac troponin T (cardiomyocytes’ specific marker) following normoxia or IH. Differentiated CMs were detected with antibodies to cardiac troponin T (green) and p-ERK1/2 or Total and ERK1/2 (red), followed by the appropriate secondary antibody. Nuclei were stained with DAPI (blue); (**B**) normoxia conditions and p-ERK1/2; (**C**) IH conditions p-ERK1/2; (**D**) normoxia conditions and Total ERK1/2; and (**E**) IH conditions and Total ERK1/2. *** *p* < 0.005; 40× magnification.

**Figure 3:**
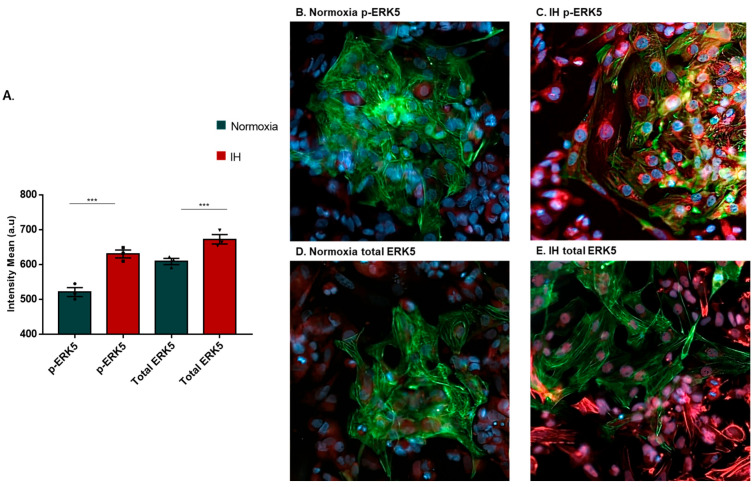
Changes in phospho ERK5 and Total ERK5 expression in hESC-CMs following IH: (**A**) Quantification of phospho-ERK5 (p-ERK5) and total ERK5 expression following Normoxia or IH. (**B**–**E**) immunostaining of p-ERK5, Total ERK5, and cardiac troponin T (cardiomyocytes’ specific marker) following normoxia or IH. Differentiated CMs were detected with antibodies to cardiac troponin T (green) and p-ERK5 or Total ERK5 (red), followed by the appropriate secondary antibody. Nuclei were stained with DAPI (blue); (**B**) normoxia conditions and p-ERK5; (**C**) IH conditions p-ERK5; (**D**) normoxia conditions and Total ERK5; and (**E**) IH conditions and Total ERK5. 40× magnification. Results are averages of 3 separate experiments done in 10 replicates, One-way ANOVA analysis *** *p* < 0.005.

In addition to ERK1/2, another related signaling pathway, ERK5, has been linked to pathological cardiac hypertrophy [22]. While ERK1/2 has been linked to hypertrophy in addition to other processes such as cell differentiation and development, ERK5 has been linked specifically to cardiac hypertrophy [23]. We were interested in determining whether phosphorylation of ERK5 (p-ERK5) would increase following IH. Similarly to the increase in phosphorylation and total ERK1/2 levels following IH (Figure 1A and Figure 2A), we also observed an increase in phosphorylation and total ERK5 levels following IH (Figure 3A). Collectively, these findings suggest that specific inhibition of ERK pathways can be of clinical relevance to alleviate sleep apnea-related cardiac damage.

### 2.3. Inhibition of ERK1/2 and ERK5 Using Specific Inhibitors

**Figure 4:**
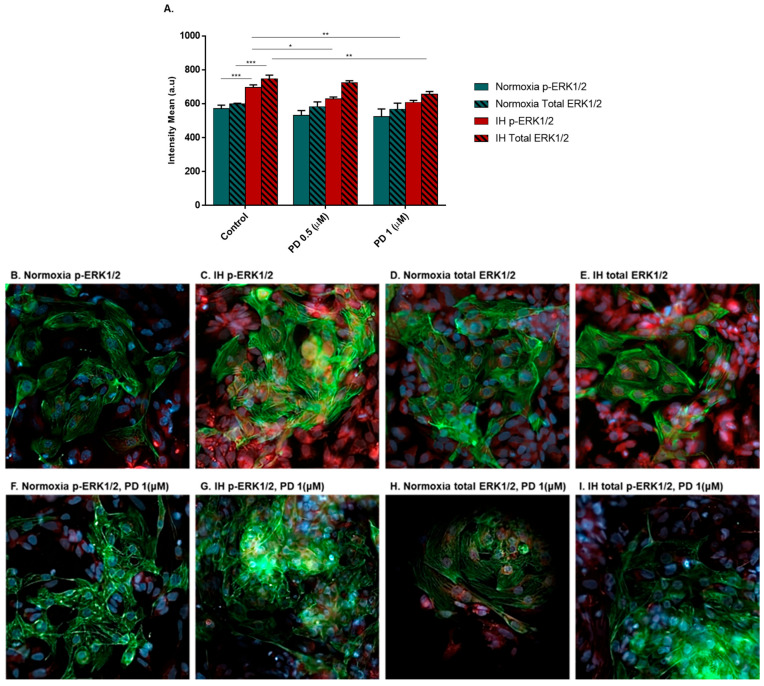
Effect of PD0325901 (PD) on p-ERK1/2 and Total ERK1/2 expression in hESC-CMs following IH: (**A**) Quantification of p-ERK1/2 and total ERK1/2 expression following normoxia (blue columns) or IH (red columns) and control or addition of inhibitor in different concentrations. Data represented as mean SEM, n = 3. Two-way ANOVA * *p* < 0.05, ** *p* < 0.01, *** *p* < 0.001. (**B**–**I**) Immunostaining of p-ERK1/2, total ERK1/2, and cardiac troponin T (cardiomyocyte-specific marker) following normoxia or IH in the presence or absence of inhibitor at different concentrations. Differentiated CMs were detected with antibodies to cardiac troponin T (green) and p-ERK1/2 or total ERK1/2 (red), followed by the appropriate secondary antibody. Nuclei were stained with DAPI (blue). (**B**) Normoxic conditions, p-ERK1/2; (**C**) IH conditions, p-ERK1/2; (**D**) Normoxic conditions, total ERK1/2; (**E**) IH conditions, total ERK1/2; (**F**) Normoxic conditions, p-ERK1/2 and PD; (**G**) IH conditions, p-ERK1/2 and PD; (**H**) Normoxic conditions, total ERK1/2 and PD; (**I**) IH conditions, total ERK1/2 and PD. 40× magnification. PD was used at two concentrations: 0.5 (µM) and 1 (µM). Only the 1 µM results are presented. Normoxia (21% O_2_, 37 °C); intermittent hypoxia (IH: 1% O_2_, 37 °C) for 12 h (60 cycles).

**Figure 5:**
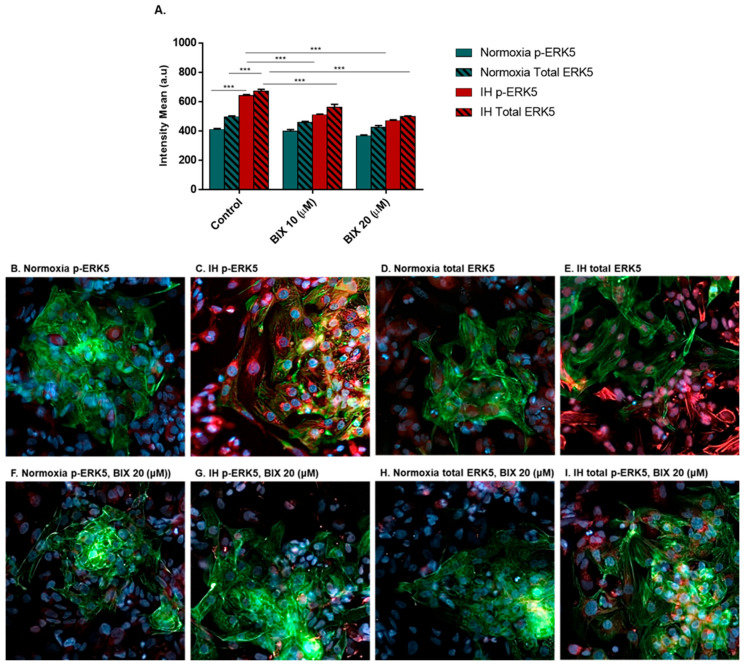
Effect of BIX02189 (BIX) on p-ERK5 and Total ERK5 expression in hESC-CMs following IH: (**A**) Quantification of p-ERK5 and total ERK5 expression following normoxia (blue columns) or IH (red columns) and control or addition of inhibitor in different concentrations. Data represented as mean SEM, n = 3. Two-way ANOVA *** *p* < 0.001. (B–I) Immunostaining of p-ERK5, total ERK5, and cardiac troponin T (cardiomyocyte-specific marker) following normoxia or IH in the presence or absence of inhibitor at different concentrations. Differentiated CMs were detected with antibodies to cardiac troponin T (green) and p-ERK5 or total ERK5 (red), followed by the appropriate secondary antibody. Nuclei were stained with DAPI (blue). (**B**) Normoxic conditions, p-ERK5; (**C**) IH conditions, p-ERK5; (**D**) Normoxic conditions, total ERK5; (**E**) IH conditions, total ERK5; (**F**) Normoxic conditions, p-ERK5 and BIX; (**G**) IH conditions, p-ERK5 and BIX; (**H**) Normoxic conditions, total ERK5 and BIX; (**I**) IH conditions, total ERK5 and BIX. 40× magnification. BIX02189 was used at two concentrations: 10 µM and 20 µM. Normoxia (21% O_2_, 37 °C); intermittent hypoxia (IH: 1% O_2_, 37 °C) for 12 h (60 cycles).

Following the increase in both ERK1/2 and ERK5 activation, we were interested in inhibiting these pathways and exploring the potential positive effects they might have. To examine the specific effect of inhibiting each pathway separately, each pathway was inhibited with a specific inhibitor at two different doses. ERK1/2 was inhibited by PD0325901 [24], and ERK5 was inhibited by BIX02189 [23]. We determined the non-toxic doses capable of causing an effect. PD0325901 caused a significant reduction in both p-ERK1/2 and its total levels (Figure 4A). BIX02189 decreased both p-ERK5 and total ERK5 levels (Figure 5A). Both inhibitors were effective in a dose-dependent manner.

### 2.4. Inhibition of ERK1/2 or ERK5 Partially Restores Beating Rate

**Figure 6:**
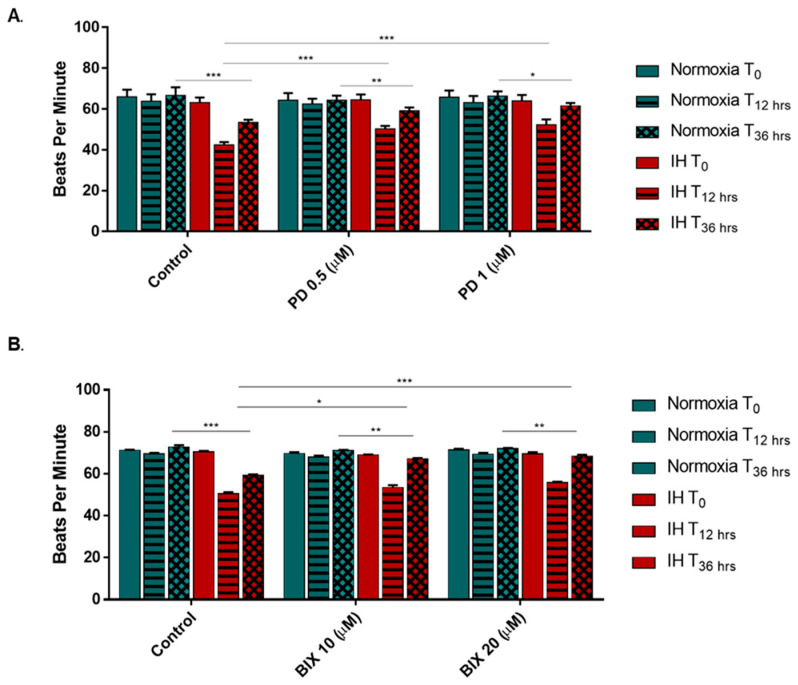
Changes in hESC-CMs beating rate following IH and ERK1/2 (PD) and ERK5 (BIX) inhibitors: Beats per minute were determined on cells under normoxic conditions (blue columns) (21% O_2_, 37 °C) or intermittent hypoxia (IH, red columns) (1% O_2_, 37 °C) for 12 h (60 Cycles). (**A**) PD0325901 (PD) was used. (**B**) BIX02189 (BIX) addition. The experiment was performed on 10 wells, in each well, 15 contracting CMs cell aggregates were counted under the microscope. Two-way ANOVA analysis * *p* < 0.05, ** *p* < 0.01, *** *p* < 0.001. Beating rate measurement following inhibitor additions, after 12 h, included media change.

Our next step was to measure a beneficial effect in the cells following the administration of the inhibitors. Since the beating rate is affected in patients during apneic episodes and can indicate a possible deterioration of the heart [25,26], we measured the beating rate of the cells before and after the experiments, as well as 24 additional hours afterwards. In Figure 6A,B, after 12 h, despite a decrease in beating rate, the decrease was less significant than without PD0325901 (PD) in a dose-dependent manner. After an additional 24 h, the recovery of the beating rate was better than in the absence of PD. Inhibition of both ERK1/2 and ERK5 partially restores the beating rate and might have a beneficial effect.

## 3. Discussion

Among the several signaling pathways implicated in OSA and its associated cardiac damage, the ERK1/2 signaling pathway stands out as one of the most extensively studied [27,28]. Elevated ERK1/2 phosphorylation has been reported in tissue samples derived from various cardiac pathologies, including heart failure and following IH [27,28,29]. Upon phosphorylation, ERK1/2 promotes the synthesis of pro-hypertrophic proteins, such as Atrial Natriuretic Peptide (ANP), Brain Natriuretic Peptide (BNP), and Myosin Heavy Chain (MHC), thereby driving cardiac remodeling and consequently compromising myocardial function and causing hypertrophy [30,31,32]. Erbin has been shown to modulate multiple signaling pathways, including ERK1/2, in which it functions as an inhibitory regulator when associated with Shoc-2 (Shoc-2) [19]. Prior studies suggest that ERK1/2 phosphorylation is reduced upon Erbin binding to Shoc-2, implying that elevated ERK1/2 activation would be accompanied by a corresponding decrease in Erbin expression [18,19]. Based on this, an increase in ERK1/2 phosphorylation concurrent with a reduction in Erbin expression was anticipated following IH exposure. Contrary to this expectation, our results showed that both proteins exhibited a significant increase following IH treatment (Figure 1A). These findings point to a more intricate relationship between ERK1/2 and Erbin than previously appreciated. Evidence from the literature indicates that Erbin’s role as an ERK1/2 modulator is contingent upon the nature of the upstream stimulus. Specifically, when the epidermal growth factor receptor (EGFR) serves as the activating trigger, Erbin functions as a suppressor of ERK1/2 activation. Conversely, when ERK1/2 activation is driven by catecholamine-induced transactivation, Erbin shifts its role to act as an enhancer of ERK1/2 signaling [33]. High levels of urinary and plasma catecholamines in OSA patients are a common occurrence that has been attributed to IH and NF-κB activation [25,34] which could explain the increase in ERK1/2 activation and Erbin following IH. Given the established involvement of ERK1/2 in IH-induced cardiac damage and the role of Erbin as an endogenous inhibitor of this pathway, it may offer valuable insight into potential strategies for the prevention of cardiac injury in the context of OSA.

Similarly to ERK1/2, the ERK5 signaling pathway has been closely associated with hypoxia, NF-κB signaling, inflammation, pathological hypertrophy, ventricular chamber thinning and dilatation, as well as heart failure [21,35,36]. While ERK1/2 remains a prominent subject of research, its broad involvement in numerous cellular processes, including apoptosis, survival, proliferation, and growth, renders it a less specific target, as many of these functions are essential for normal cellular homeostasis [37]. In contrast, ERK5 activation has been more selectively implicated in pathological conditions, which prompted its investigation in the present study. The results demonstrated a significant increase in the phosphorylation of both ERK1/2 and ERK5, as well as their total protein levels, following IH exposure (Figure 2A and Figure 3A), consistent with our hypothesis. The observed activation of these signaling pathways opens avenues for their potential modulation as a therapeutic strategy to mitigate IH-induced cardiac damage.

To evaluate the potential beneficial effects of ERK1/2 and ERK5 signaling pathway inhibition, pathway-specific inhibitors were employed for each respective pathway, taking into account the fact that certain inhibitors are capable of suppressing both pathways simultaneously at specific concentrations [20,23,38]. To ensure the absence of cytotoxic effects, two doses of each inhibitor were evaluated, ERK1/2 inhibition was achieved using PD0325901, which exerts its effect by binding to MEK1/2 and thereby preventing downstream ERK1/2 activation [24]. The higher concentration of PD0325901 produced a significant reduction in ERK1/2 phosphorylation compared to the lower dose. This dose-dependent effect was not similarly reflected in total ERK1/2 protein levels; however, the higher concentration did yield a significant reduction in total ERK1/2 protein levels (Figure 4A). ERK5 inhibition was achieved using BIX02189, which exerts its effect by binding to MEK5, thereby preventing downstream ERK5 activation [23,38]. Analysis of the data revealed a significant dose-dependent reduction in both ERK5 phosphorylation levels and total ERK5 protein levels following inhibitor treatment (Figure 5A).

The subsequent aim of the study was to investigate the potential beneficial effects of ERK1/2 or ERK5 inhibition on a physiological parameter known to be compromised in OSA. Patients with sleep apnea frequently experience cardiac rhythm disturbances, including tachycardia, characterized by an abnormally elevated resting heart rate, and bradycardia, defined as a resting heart rate below 60 beats per minute [26,39]. Such disturbances may trigger a deterioration of cardiac physical function as an early consequence of OSA, one that has been shown to be unresponsive to CPAP treatment [40,41]. Given the significant reduction in beating rate observed in the present model following IH exposure, cells were treated with the respective inhibitor at the previously described doses, and beating rate was measured at baseline, following 12 h of IH, and after an additional 24 h of recovery under normoxic conditions and media change.

ERK1/2 inhibition attenuated the IH-induced decline in beating rate, with a statistically significant partial restoration observed at both doses, albeit without a clear dose-dependent relationship. Upon assessment of the beating rate following the additional 24-h recovery period, IH-exposed cells demonstrated a dose-dependent restoration of the beating rate, approaching values comparable to those exhibited by cells maintained under normoxic conditions (Figure 6A).

ERK5 inhibition attenuated the IH-induced decline in beating rate, with a statistically significant partial restoration observed at both doses in a dose-dependent manner. Upon assessment of beating rate following the additional 24-h recovery period, IH-exposed cells demonstrated partial restoration of beating rate without a clear dose-dependent relationship, probably due to dose saturation (Figure 6B).

The present study demonstrated that IH induces activation of the ERK1/2, Erbin and ERK5 signaling pathways in hESC-CMs, with both ERK1/2 and ERK5 phosphorylation significantly elevated following IH exposure. The unexpected concurrent increase in Erbin expression alongside ERK1/2 activation suggests a more complex regulatory relationship than previously described, potentially driven by catecholamine-induced signaling in the context of OSA. Targeted inhibition of ERK1/2 using PD0325901 and ERK5 using BIX02189 attenuated the IH-induced decline in beating rate, with partial to significant restoration observed at both doses and following normoxic recovery. Collectively, these findings suggest that modulation of the ERK1/2 and ERK5 signaling pathways may represent a promising therapeutic strategy to mitigate IH-induced cardiac dysfunction in OSA patients.

Our in vitro model recapitulates, at the cellular level, the effects of intermittent hypoxia that characterizes OSA. While innovative in its design, the system shares inherent limitations with other in vitro approaches: it lacks the physiological extracellular milieu present in intact tissue, and the cardiomyocytes remain immature, precluding the formation of a fully differentiated, functional organ. Furthermore, OSA-related cardiac pathology in patients develops over years of cumulative exposure, whereas our experimental conditions examine effects over a compressed timeframe of hours to days. These constraints necessarily should be considered in the translational scope of our conclusions.

Nevertheless, the use of human embryonic stem cell–derived cardiomyocytes affords a physiologically relevant platform for interrogating the molecular, cellular, and functional consequences of OSA on cardiac cells. Elucidating the signaling cascades activated by intermittent hypoxia and their impact on cardiomyocyte function may ultimately advance our understanding of the progressive cardiac damage associated with this highly prevalent condition.

## 4. Materials and Methods

### 4.1. Antibodies

Anti-Cardiac Troponin T antibody [1C11], ab8295, Abcam, Cambridge, UK.

Erbin Polyclonal Antibody, bs-14619R, Bioss, Woburn, MA, USA.

Phospho-p44/42 MAPK (Erk1/2) (Thr202/Tyr204) (D13.14.4E) XP^®^ Rabbit mAb, 4370, Cell Signaling Technology, Danvers, MA, USA.

Anti-ERK1 + ERK2 antibody [EPR17526], ab184699, Abcam, Cambridge, UK.

p44/42 MAPK (Erk1/2) Antibody, 9102, Cell Signaling Technology, MA, USA.

Phospho-Erk5 (Thr218/Tyr220) Antibody, 3371, Cell Signaling Technology, MA, USA.

Erk5 (D3I5V) Rabbit mAb, 12950, Cell Signaling Technology, MA, USA.

Goat anti-Mouse IgG (H+L) Highly Cross-Adsorbed Secondary Antibody, Alexa Fluor 488 [A-11029] and Goat anti-Rabbit IgG (H+L) Highly Cross-Adsorbed Secondary Antibody, Alexa Fluor 633 [A-21071], Invitrogen, Thermo Fisher Scientific, Waltham, MA, USA.

Vectashield Mounting Medium with DAPI: Vector Laboratories, Burlingame, CA, USA.

### 4.2. Cardiomyocytes (CMs) Differentiation

WA-09 cells, human embryonic stem cells were obtained from Dr. Rivki Ofir and differentiated to cardiomyocytes as described before [14]. Briefly, hES cells were maintained on Matrigel in NutriStem medium, dissociated into monocultures cells with Accutase solution [14] at 37 °C, for 2 min and seeded to a density of 8.5 × 10^5^ cells/well (in 12-well plates). The amount of Matrigel coating was doubled as recommended by the manufacturer. The cells were cultured in NutriStem supplemented with the ROCK inhibitor, Y-27632 (5 μM) for 1 day (day −5), then in NutriStem medium, which was changed daily. After 5 days, at day 0, the cells were supplemented with the Glycogen Synthase Kinase-3 (Gsk3) inhibitor CHIR99021 (6 μM) in RPMI that contains 1:50 B27 without insulin for 1 day. On day 1, the medium was removed and replaced with fresh medium and Gsk3 inhibitor for 1 day. On day 2, the medium was changed to RPMI/B27 without insulin for another 1 day. Next (day 3), the Wingless and Int-1 (Wnt) production-2 inhibitor, IWP2 (3 μM), was added for an additional 1 day and then was removed (day 4) by changing the medium to RPMI/B27 without insulin. The medium was changed every day, but not on day 6. Finally, on day 7, the cells were maintained in RPMI/B27 (1:50) supplement medium that was changed every day. First, spontaneous cell beatings appeared on days 7 to 11 of differentiation. The cells were kept in 5% CO_2_ in a 37 °C heated incubator. Cells were transferred to 96 wells (50 × 10^3^ cells/well) for evaluation of different parameters.

### 4.3. Inhibitors Treatment

In order to induce selective inhibition of ERK1/2 (PD0325901) or ERK5 (BIX 02189, 4842, R&D SYSTEMS, Minneapolis, MN, USA), a specific inhibitor for each was added to the media immediately prior to induction of normoxia or IH. Each inhibitor was dissolved in 0.1% Dimethyl sulfoxide (DMSO).

### 4.4. Hypoxia Induction by Using the “OxyCycler System”

Intermittent hypoxia (IH) exposure was conducted using a custom-designed computer-controlled incubator chamber connected to BioSpherix OxyCycler (Biospherix, Redfield, NY, USA). Cells were maintained at 37 °C at 5% CO_2_ in the hypoxic chamber in which O_2_ levels were alternated between 1% for 8 min and ~21% for 4 min. Cells in the control group were maintained in normoxic conditions (~21% O_2_ and 5% CO_2_) throughout the experiment. Cells were exposed to normoxia or intermittent hypoxia for 12 h, a total of 60 cycles in 12 h.

### 4.5. Beating Rate Measurement

Beating rate was determined initially on cells under normoxia at time 0 and after 12 h of IH or normoxic conditions. Follow-up measurements were taken after media changes and incubation under normoxic conditions. Beating cells in different areas of the well were scored for the number of cell-contractions/minute under the microscope. A total of 10 contracting cell aggregates were counted/well/minute; 15 wells were scored per experiment. Each experiment was repeated three times. Beating rate measurement following inhibitor additions after 12 h included media change. Statistical significance was determined.

### 4.6. Cell Discoverer 7—Zeiss

In order to quantify the expression of a specific protein in cardiomyocytes (troponin-positive cells), we measured the relative amount of the specific protein by immunofluorescence. A total of 50,000 differentiated CM cells were plated per well of a 96-well plate and allowed to attach for 24 h at 37 °C. Then the cells were transferred to an OxyCycler incubator (Biospherix, Redfield, NY, USA). Cells were maintained at 37 °C at 5% CO_2_ in the hypoxic chamber in which O_2_ levels were alternated between 1% for 8 min and ~21% for 4 min, a total of 60 cycles in 12 h. Cells in the control group were maintained in normoxic conditions (~21% O_2_ and 5% CO_2_) throughout the experiment. At the end of the experiment, the cells were washed twice with Phosphate-Buffered Saline (PBS) and fixed with 4% paraformaldehyde in PBS at room temperature (RT) for 20 min. Cells were washed twice again in PBS before permeabilization in 3% Fetal Bovine Serum (FBS) in PBS and blocked with 0.1% Triton X100 in 3% FBS and PBS for 60 min at RT. Cells were washed twice with 3% FBS in PBS before staining with an antibody to cardiac Troponin T (cTnT), a known CM marker troponin [42], and the desired primary antibody (p65, p50 etc.) in 3% FBS and 0.1% Triton X100 in PBS for overnight. The cells were then stained with a secondary antibody AF488 (green) and AF 688 (red) for overnight and DAPI (blue-nuclear) staining for 30 min, followed by 3× washing with 0.1% Triton X100 in 3% FBS and PBS. The cells were imaged with the Cell Discoverer 7—Zeiss system (Jena, Germany) at 40× magnification. Image Acquisition: Using a 20× magnification, images were taken with the Zeiss Cell Discoverer 7 Imaging System. The picture capture program was Zen Blue 3.7.

Protein Expression Quantification: Analysis of the pictures was performed through the ImageJ software version 1.54e, https://imagej.net/ij/, accessed on 1 April 2025. In which we can define parameters such as selective fluorescence quantitative scoring of cardiomyocytes (green cells), and the antigen of interest in the nucleus or the cytoplasm (red), specifically in these cells.

### 4.7. Statistical Analysis

Values were expressed as mean ± SEM. Statistical analysis was performed using Prism 7.0 (GraphPad Software, San Diego, CA, USA). Comparisons between normoxia and IH groups were performed using an unpaired Student’s *t*-test or ANOVA analysis. The specific tests that were used are mentioned in the legend of each figure. The criteria for significance were set at *p* < 0.05. Unless otherwise stated, p-values were displayed graphically as follows: * *p* < 0.05, ** *p* < 0.001, *** *p* < 0.0001. 

## Data Availability

The original contributions presented in this study are included in the article. Further inquiries can be directed to the corresponding author.
